# High-dose Vitamin C injection ameliorates against sepsis-induced myocardial injury by anti-apoptosis, anti-inflammatory and pro-autophagy through regulating MAPK, NF-κB and PI3K/AKT/mTOR signaling pathways in rats

**DOI:** 10.18632/aging.205735

**Published:** 2024-04-19

**Authors:** Ya-Nan Cui, Na Tian, Yan-Hai Luo, Ji-Jun Zhao, Cheng-Fei Bi, Yi Gou, Jia Liu, Ke Feng, Jun-Fei Zhang

**Affiliations:** 1 Medical Records and Statistics Room, General Hospital of Ningxia Medical University, Yinchuan, Ningxia 750000, China; 2 Department of Emergency Medical, General Hospital of Ningxia Medical University, Yinchuan, Ningxia 750000, China; 3 Department of Pathology, General Hospital of Ningxia Medical University, Yinchuan, Ningxia 750000, China; 4 School of Clinical Medicine, Ningxia Medical University, Yinchuan, Ningxia 750000, China

**Keywords:** High-dose Vitamin C, sepsis-induced myocardial injury, apoptosis, autophagy, inflammatory, MAPK, NF-κB and PI3K/AKT/mTOR signaling pathways

## Abstract

Aims: This study aimed to evaluate the effects of VC on SIMI in rats.

Methods: In this study, the survival rate of high dose VC for SIMI was evaluated within 7 days. Rats were randomly assigned to three groups: Sham group, CLP group, and high dose VC (500 mg/kg i.v.) group. The animals in each group were treated with drugs for 1 day, 3 days or 5 days, respectively. Echocardiography, myocardial enzymes and HE were used to detect cardiac function. IL-1β, IL-6, IL-10 and TNF-α) in serum were measured using ELISA kits. Western blot was used to detect proteins related to apoptosis, inflammation, autophagy, MAPK, NF-κB and PI3K/Akt/mTOR signaling pathways.

Results: High dose VC improved the survival rate of SIMI within 7 days. Echocardiography, HE staining and myocardial enzymes showed that high-dose VC relieved SIMI in rats in a time-dependent manner. And compared with CLP group, high-dose VC decreased the expressions of pro-apoptotic proteins, while increased the expression of anti-apoptotic protein. And compared with CLP group, high dose VC decreased phosphorylation levels of Erk1/2, P38, JNK, NF-κB and IKK α/β in SIMI rats. High dose VC increased the expression of the protein Beclin-1 and LC3-II/LC3-I ratio, whereas decreased the expression of P62 in SIMI rats. Finally, high dose VC attenuated phosphorylation of PI3K, AKT and mTOR compared with the CLP group.

Significance: Our results showed that high dose VC has a good protective effect on SIMI after continuous treatment, which may be mediated by inhibiting apoptosis and inflammatory, and promoting autophagy through regulating MAPK, NF-κB and PI3K/AKT/mTOR pathway.

## INTRODUCTION

Although the international consensus on sepsis defines the current concept of sepsis, the current definition of sepsis is still controversial. It has become a common understanding that sepsis can cause damage to vital organs and further aggravate the condition [1–3]. The heart is an important organ vulnerable to attack after the onset of sepsis, and studies have shown that the mortality of patients with myocardial damage caused by sepsis is significantly increased [3, 4]. However, the mechanism by which sepsis-induced myocardial injury (SIMI) remains unclear [5, 6]. Our previous studies have also demonstrated that autophagy and apoptosis play key roles in the occurrence and development of SIMI [7]. The PI3K/AKT/mTOR [8], MAPK [9] and NF-κB [10] and pathways regulate various biological responses, including proliferation, autophagy, inflammation, and apoptosis [11–15]. Inhibition of PI3K/AKT/mTOR signaling pathway can enhance autophagy and thus reduce oxidative stress and apoptosis in LPS -induced sepsis [16]. Additionally, inhibition of NF-κB and MAPK mediated the anti-inflammatory and anti-apoptotic effects, thereby protecting the heart from LPS-induced injury [17]. These results suggest that the PI3K/AKT/mTOR, MAPK and NF-κB signaling pathways can be used to treat SIMI.

**Figure 1:**
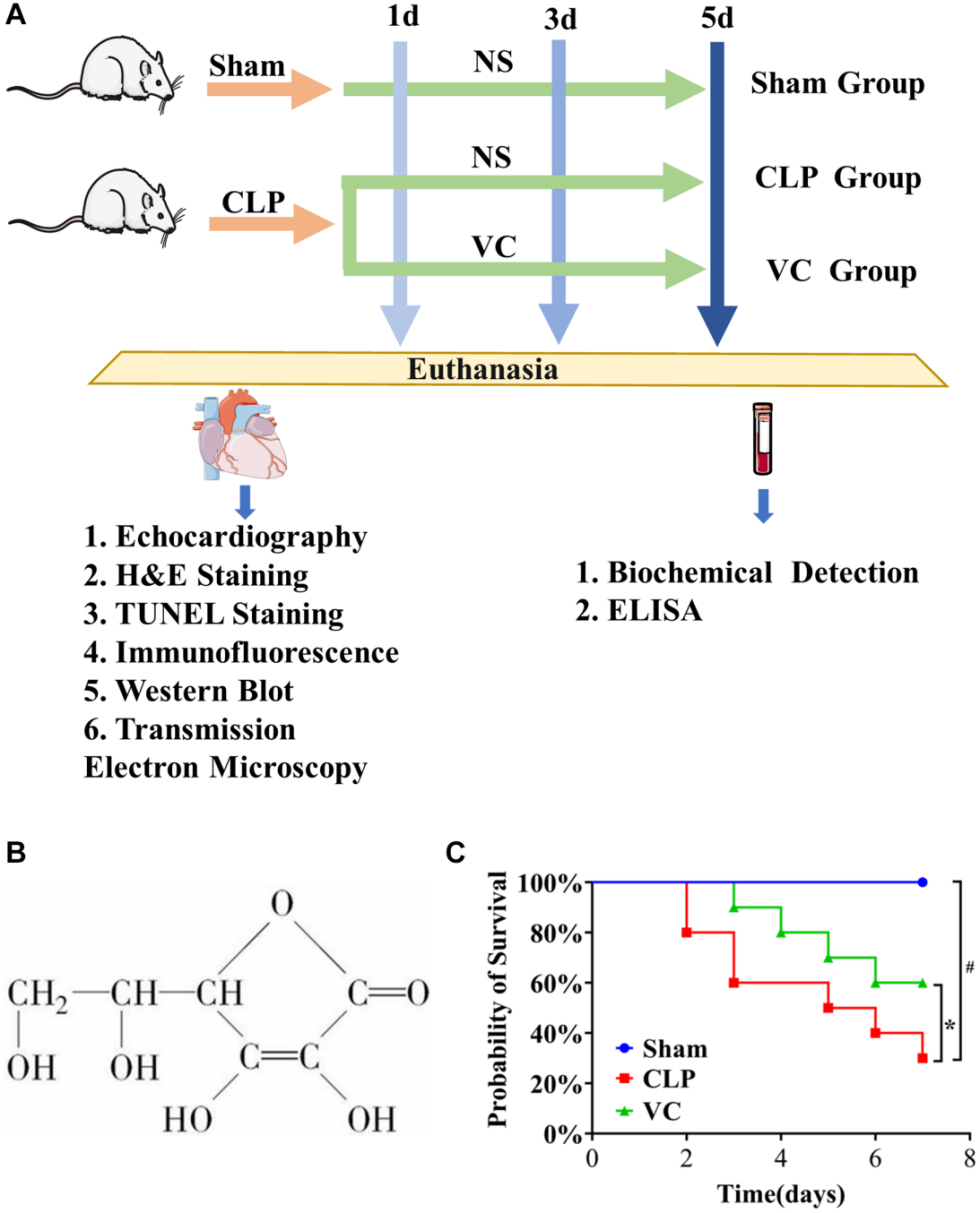
(**A**) The whole process of drug administration in animal experiments. (**B**) The chemical structure of VC. (**C**) The survival rate of high dose VC for SIMI was evaluated within 7 days.

As a micronutrient, Vitamin C (Figure 1B) has shown great potential in the adjuvant therapy of critically ill patients [18, 19]. A study showed that VC in sepsis attenuated oxidative stress and inflammation, improved vasopressor synthesis, enhanced immune cell function and improved endovascular function, which may become standard of care for the treatment of sepsis [20]. But several studies have shown that taking VC has no beneficial effect on sepsis patients [21, 22]. Therefore, this paper is to study the efficacy and specific mechanism of high dose VC alone in sepsis.

In this study, SIMI models 1d, 3d and 5d after surgery were constructed by CLP, and high-dose VC was given for treatment, so as to observe the effect of VC on SIMI after continuous treatment and explore the specific mechanism of action.

## RESULTS

### High-dose VC improved the survival rate and viability in CLP-induced sepsis rats

To evaluate the effect of VC on the survival of mice after CLP, 7-day survival was assessed. Figure 1C shows that the survival rate in the CLP group dropped by approximately 20% at 2 days after surgery and continued to decline sharply, while the survival rate in the VC group dropped by about 10% at 3 days after surgery. The mortality rates at 7 days were 0%, 70% and 40% in the sham, CLP and VC groups, respectively. Compared with CLP group, the mortality of rats in VC group was significantly reduced (*p* < 0.05). VC prolonged the survival time. In addition, the results also showed that CLP-induced septic rats did not develop lethality within 2 days, and VC prolonged the survival time of lethal infection with CLP. The food intake, water intake, activity level and mental state of rats in the sham operation group returned to normal soon after operation. The rats in CLP group showed reduced water intake, reduced activity, eye oozing blood, poor mental state, slow reaction, inverted dorsal hair, tarnish, and reduced defecation, while the rats in VC group showed increased water intake, increased activity, mental activity, and no eye oozing blood. This result preliminarily confirmed the protective effect of VC on septic rats. Based on this result, high-dose VC (500 mg/kg) was continued in the subsequent study.

### High-dose VC relieved the cardiac function in CLP-induced sepsis rats

To assess the effect of the cardiac function after CLP, Echocardiography, H&E Staining, transmission electron micrographs and the serum necrosis makers in rats were assessed at 1 day, 3 days and 5 days.

**Figure 2:**
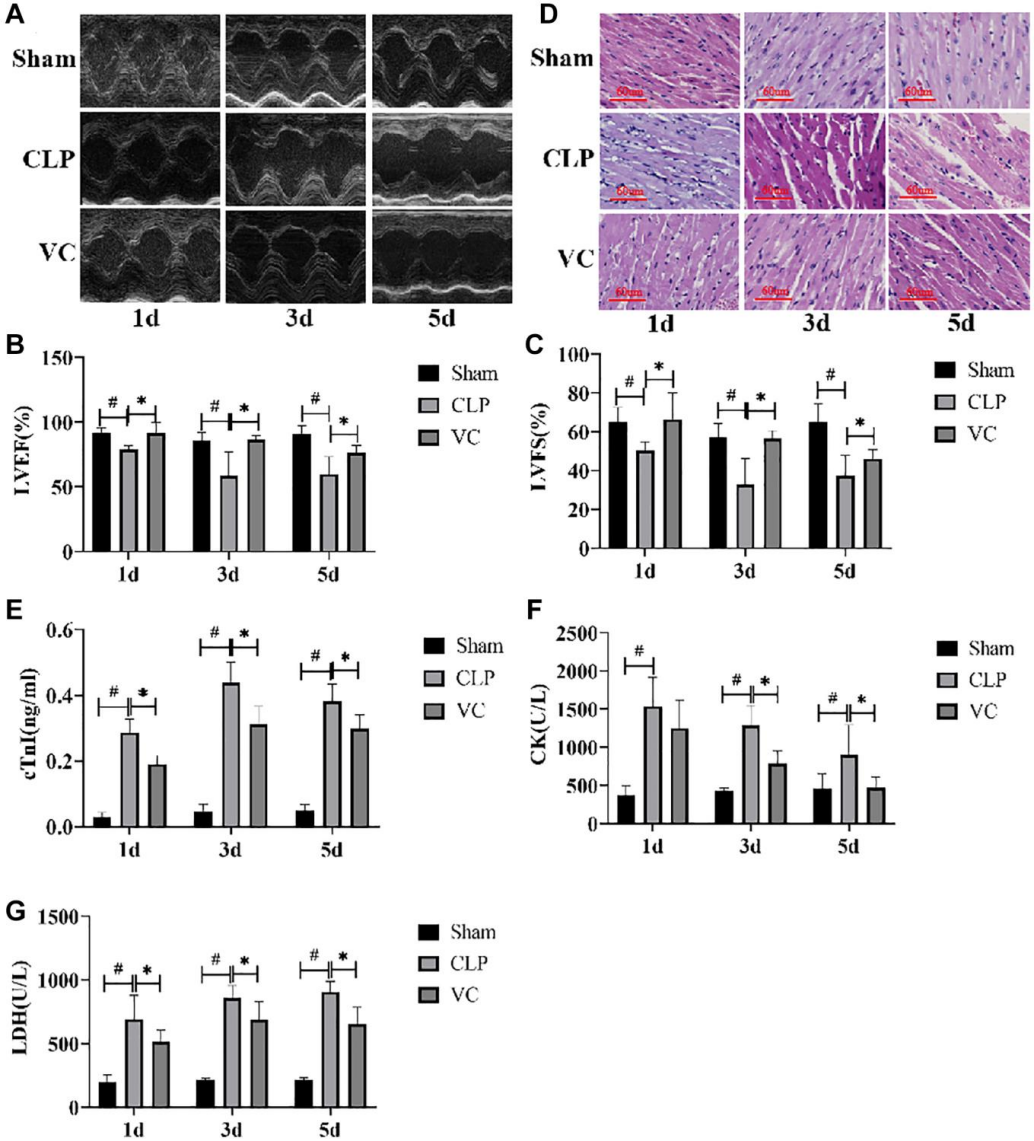
**High-dose VC relieved the myocardial dysfunction in CLP-induced sepsis rats at 1d, 3d and 5d.** (**A**–**C**) Echocardiographic image of each group of rats. LVEF and LVFS were quantified via echocardiography. (**D**) Myocardial injury was detected by H&E staining. (**E**–**G**) Myocardial injury markers in serum, cTnI, CK, and LDH were detected to visualize myocardial injury using a fully automated biochemical analyzer. Data are expressed as mean ± SD (at least *n* = 6/group), ^#^*p* < 0.05 (vs. Sham group), ^*^p < 0.05 (vs. CLP group).

To investigate whether high-dose VC relieved cardiac function in rats with SIMI, we performed LVEF and LVFS by Echocardiography at 1 day, 3 days and 5 days. As in Figure 2A–2C, LVEF and LVFS in CLP group were significantly lower than those in sham group (*p* < 0.05), while administration of high-dose VC increased LVEF and LVFS in dose-dependent manner, indicating that high-dose VC cloud improve cardiac function in rats with sepsis.

H&E staining indicated that striation of the myocardium was clear, the cells were arranged neatly, and the myocardial structure was normal in the sham group at different time gradients. On the contrary, the CLP group had obvious inflammatory cell infiltration, abnormal myocardial cell structure, interstitial edema, unclear myocardial fiber texture and local necrosis. With the prolongation of exposure time to CLP, the degree of cardiac tissue damage in septic rats was gradually aggravated. High-dose VC can significantly improve the above obstacles, indicating that high-dose VC cloud improve myocardial histopathological changes in sepsis rats. (Figure 2D).

We further evaluated the expression of myocardial enzymes in rats. Figure 2E–2G showed that the expression levels of myocardial enzymes (cTn, CK, and LDH) were significantly increased with the increase of postoperative time of CLP compared with Sham group, while the expression levels of myocardial enzymes could be decreased after VC treatment. It is worth noting that there is no significant difference between CK level at 1 day and 5 day and LDH level at 1 day. The change of CK level in CLP group and VC group was time dependent, but the other two indexes were not.

These results demonstrated that Cardiomyocytes in CLP-induced sepsis rats showed obvious myocardial injury characteristics after polymicrobial sepsis stimulation in a time-dependent manner, and high-dose VC has a protective effect on SIMI in rat.

### High-dose VC inhibited the myocardial cell apoptosis in CLP-induced sepsis rats

**Figure 3:**
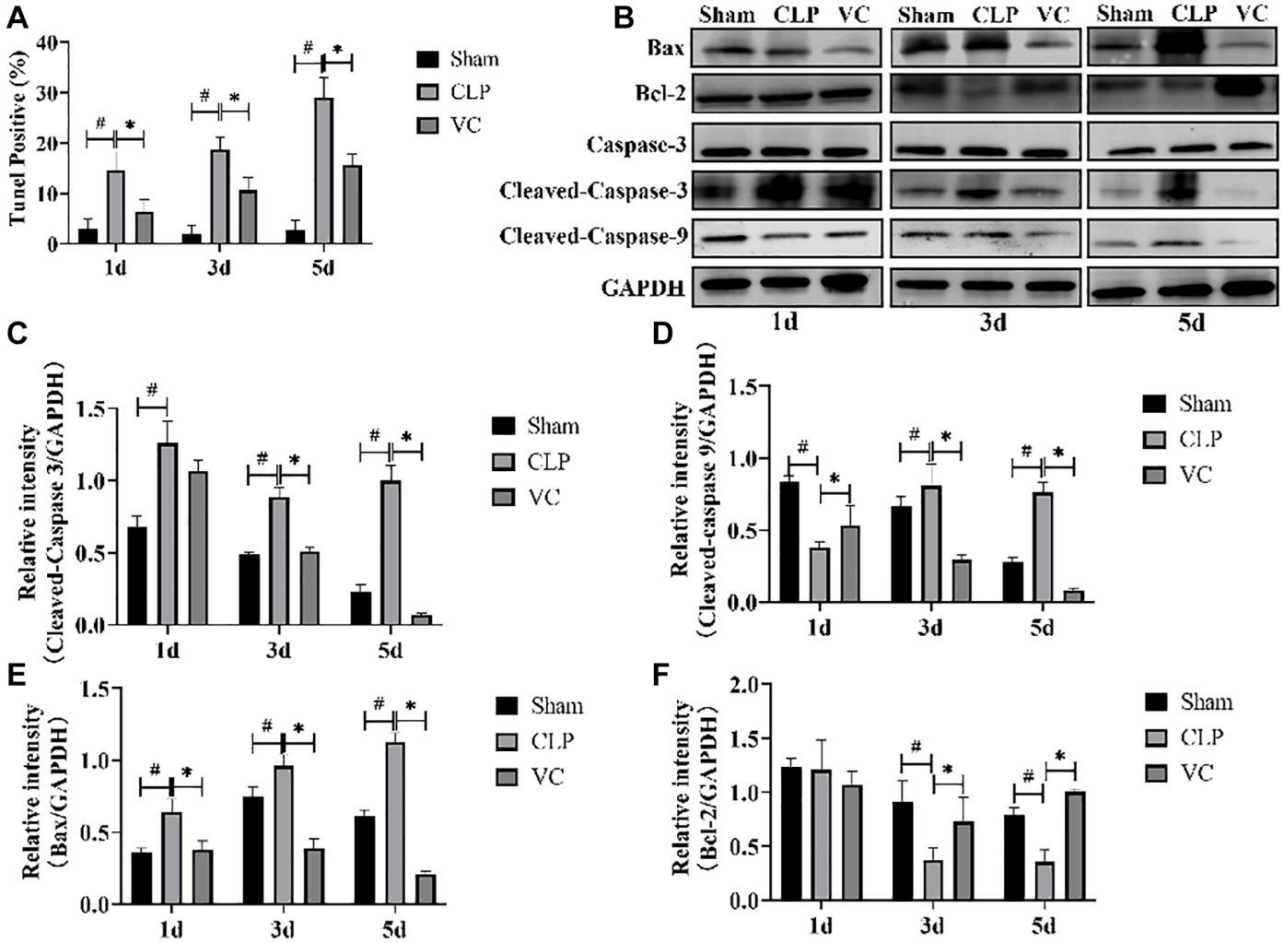
**High-dose VC suppressed the cardiomyocyte apoptosis in CLP-induced sepsis rats at 1d, 3d and 5d.** (**A**) Apoptotic cells were detected by TUNEL staining. (**B**–**F**) Representative images of Caspase-3 activity, Cleaved-caspase 3, Cleaved-caspase 9, and Bax and Bcl-2 expression were examined by western blot and the fold activation data analysis. Data are expressed as mean ± SD (at least *n* = 6/group), ^#^*p* < 0.05(vs. Sham group), ^*^p < 0.05 (vs. CLP group).

The extent of apoptosis could prove the injury characteristics of cardiomyocytes. Then, we investigated whether administration of therapy of high-dose VC could influence apoptosis process during sepsis at 1 day, 3 days and 5 days. Caspase-3 activity, Cleaved-caspase 9, Cleaved-caspase 3, Bcl-2 and Bax expression were examined to confirm apoptosis using western blot. As Figure 3B–3F, compared with the sham group, Cleaved-caspase 9, Cleaved-caspase-3 (except for 1d) and Bax expression were increased in myocardial tissue of CLP group rats, whereas these were elevated by therapy of high-dose VC (Cleaved-caspase-3 and Cleaved-caspase 9 except for 1d). Conversely, therapy of high-dose VC increased the expression levels of Bcl-2 (except for 1d). Following, TUNEL staining results also showed that VC staining decreased cardiomyocyte apoptosis rate (*p* < 0.05) (Figure 3A). The above results indicated that CLP led to cardiomyocyte apoptosis in a time-dependent manner and high-dose VC attenuated septic rats’ cardiomyocyte apoptosis.

### High-dose VC attenuated the inflammatory response in CLP-induced sepsis rats

**Figure 4:**
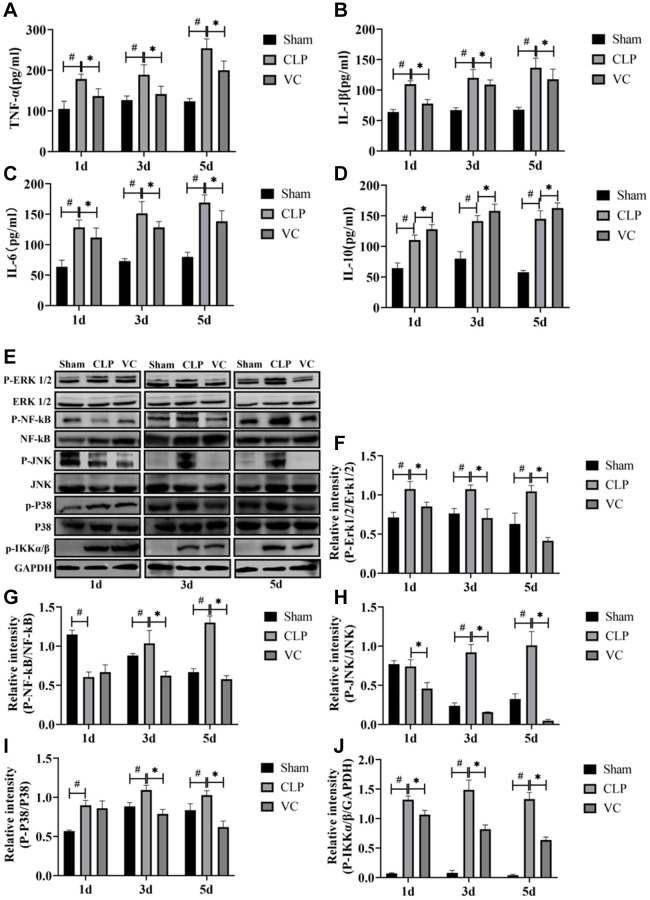
**High-dose VC attenuated the inflammatory response in CLP-induced sepsis rats at 1d, 3d and 5d.** (**A**–**D**) The serum inflammatory cytokines (i.e., TNF-α, IL-1β, IL-6, and IL-10) in serum were measured by ELISA. (**E**–**J**) Representative images of phosphorylation levels of P38, Erk1/2, JNK, NF-κB and IKK α/β were examined by western blot and the fold activation data analysis. Data are expressed as mean ± SD (at least *n* = 6/group), ^#^*p* < 0.05 (vs. Sham group), ^*^p < 0.05 (vs. CLP group).

Figure 4A–4D shows that with the increase of time after CLP, the expression of inflammatory factors (IL-6, IL-1β, IL-10 and TNF-α) in the serum of rats is significantly increased, and the expression level of such inflammatory factors (IL-6, IL-1β and TNF-α) can be reversed to a certain extent after VC treatment, in addition, VC further improves the expression level of IL-10.

To elucidate whether the inhibition of the secretion of inflammatory mediators by high-dose VC was mediated through MAPK and NF-κB signaling pathways, MAPK (i.e., P38, Erk1/2 and JNK) and NF-κB (i.e., NF-κB and IKK α/β) signaling molecules at 1 day, 3 days and 5 days were measured by western blot, respectively. Figure 4E–4J indicated that compared to the sham group, the CLP induced a strong phosphorylation of P38, Erk1/2, JNK, NF-κB and IKK α/β (JNK and NF-κB except for 1d) in a time-dependent manner (*p* < 0.05), and these (NF-κB except for 1d) were decreased by high-dose VC (*p* < 0.05). The five proteins in VC group seemed to be obviously lower than these in CLP group (*p* < 0.05).

### High-dose VC enhanced the cardiomyocyte autophagy in CLP-induced sepsis rats

**Figure 5:**
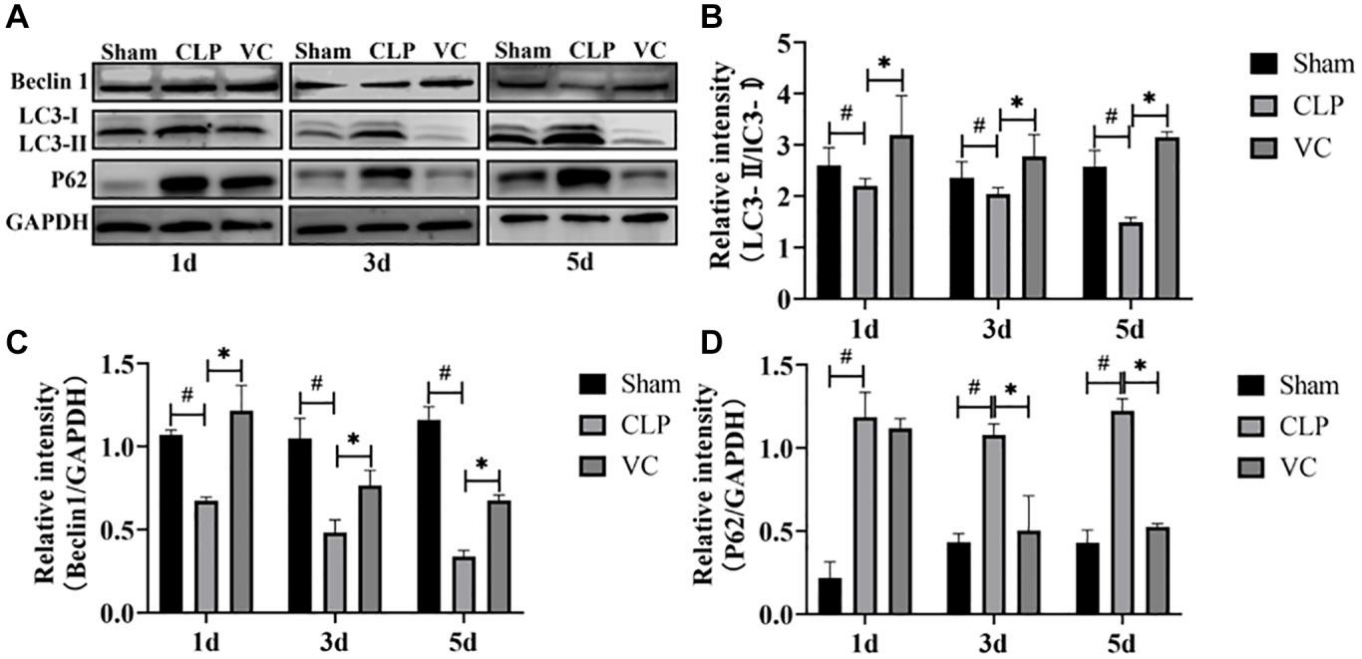
**High-dose VC enhanced the cardiomyocyte autophagy in CLP-induced sepsis rats at 1d, 3d and 5d.** (**A**–**D**) Representative images of LC3-II/LC3-I ratio and the expression of the proteins Beclin-1 and P62 were examined by western blot and the fold activation data analysis. Data are expressed as mean ± SD (at least *n* = 6/group), ^#^*p* < 0.05 (vs. Sham group), ^*^p < 0.05 (vs. CLP group).

The ratio of LC3II to LC3I was positively correlated with autophagosome activity. Beclin-1 and LC3-II are regarded as a negative regulator of autophagic activity. Conversely, P62 plays a crucial role inhibiting autophagy. To investigate the effect of high-dose VC on cardiomyocyte autophagy during sepsis, the protein expression of Beclin-1, and P62 and the LC3-II/LC3-I ratio in different groups were measured by western blot at 1 day, 3 days and 5 days. Figure 5A–5D showed that the expression level of the protein LC3-II/LC3-I ratio and Beclin-1 in a time-dependent manner were importantly decreased, while compared with the sham group, the expression level of P62 in the myocardium of septic rats was significantly increased at different time points. But, compared with CLP group, high dose VC treatment significantly increased myocardial LC3-II/LC3-I ratio and Beclin-1 protein expression level, and decreased myocardial P62 expression. These results showed that CLP led to the diminution of cardiomyocyte autophagy and high-dose VC boosted septic rats’ autophagy.

### High-dose VC inhibited the PI3K/AKT/mTOR signaling pathway in CLP-induced sepsis rats

**Figure 6:**
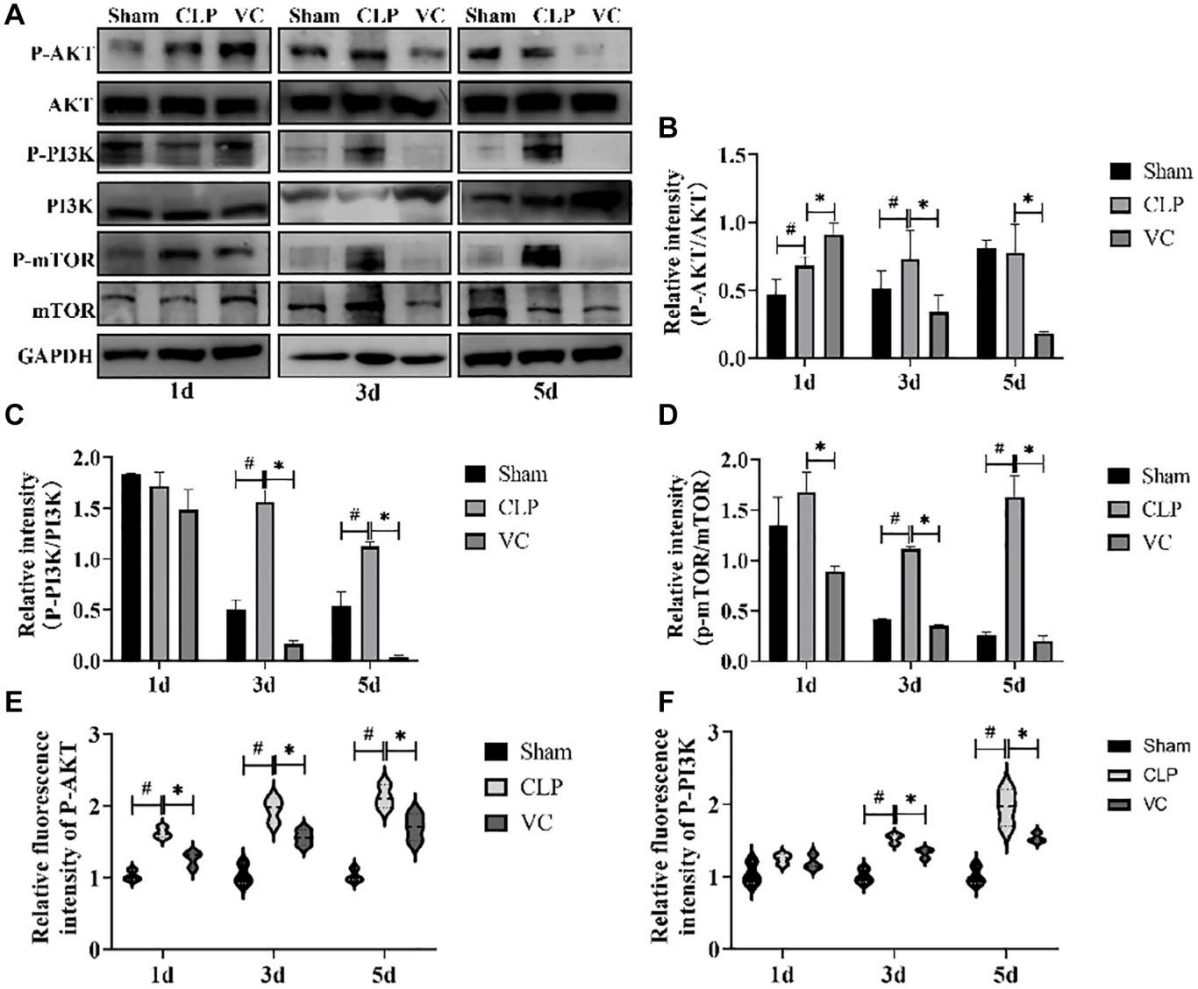
**High-dose VC inhibited the PI3K/AKT/mTOR signaling pathway in CLP-Induced sepsis rats.** (**A**–**D**) Representative images of the phosphorylation of PI3K, AKT and mTOR were examined by western blot and the fold activation data analysis. (**E**, **F**) The expression of the proteins p-Akt and p-PI3K was examined by immunofluorescence assay. Data are expressed as mean ± SD (at least *n* = 6/group), ^#^*p* < 0.05 (vs. Sham group), ^*^p < 0.05 (vs. CLP group).

To further investigate the effect of high-dose VC on autophagy and apoptosis pathways, the effect of PI3K/AKT/ mTOR signal pathway in high-dose VC-treated CLP-induced sepsis rats was assessed (Figure 6A–6D). Western blot results demonstrated that CLP increased the phosphorylation of PI3K (except 1 days), AKT (except 5 days) and mTOR in different time points. Conversely, high-dose VC obviously decreased phosphorylated PI3K, AKT (except 1 days) and mTOR in CLP-induced sepsis rat cardiomyocytes at three points in time (*p* < 0.05), but it did not affect the phosphorylation of PI3K and AKT at 1 day. None of the groups affected the expression levels of total PI3K, AKT or mTOR. Further immunofluorescence experiments of P-PI3K (Figure 6F) and P-AKT (Figure 6E) were consistent with the results of Western blot. The above results suggested that over time, high-dose VC protects SIMI in rats by gradually enhancing the inhibition of PI3K/AKT/mTOR signaling pathway.

## DISCUSSION

Vitamin C (VC) has gone through centuries from knowledge, isolation and use [23]. With the in-depth study of VC, VC as an antioxidant is known to us, and has become an indispensable nutrient for the human body [24–26]. The effectiveness and safety of intravenous VC in the treatment of sepsis has been controversial. Several meta-analysis studies [27–29] consistently showed that VC administration was not associated with lower mortality, but one of the studies also showed that patients may benefit if VC is administered for more than 3–4 days [28]. Other studies have found that high-dose IV VC monotherapy or in combination with standard therapy appeared safe, may be associated with improved mortality [18, 30]. The present study revealed that after 7 days of continuous treatment with high-dose VC, the mortality of sepsis rats decreased significantly, which may provide a reasonable start for sepsis treatment. Of course, there were some limitations to the results due to the small number of animals. Therefore, further studies are needed to identify subgroups of patients who may benefit from intravenous supplementation with high dose VC. What’s more, pathologically, VC deficiency presents as scurvy, and subnormal plasma VC concentration is negatively correlated with the incidence of multiple organ failure in patients with sepsis [31]. It was found that VC-deficient mice were more likely to develop multiple organ dysfunction caused by sepsis, and intravenous VC administration alleviated the damage (lung, kidney, liver) [32]. It has been reported that VC has a beneficial effect on the heart in patients with sepsis [33]. Clinical trials have found that intravenous VC infusion is helpful in the treatment of multiple organ failure [34]. There was also data suggesting that VC alleviated LPS-induced SIMI [19]. We also studied the therapeutic effect of VC on CLP-induced SIMI in rats. First, high-dose VC significantly improved the LVEF and LVFS after CLP in sepsis rats. Second, high-dose VC significantly relieved noticeable inflammatory cell infiltration, indistinct myocardial fiber texture and local necrosis in septic rats. Finally, high-dose VC significantly improved the myocardial injury markers (cTnI, CK, and LDH) in the serum of sepsis rats. Generally speaking, High-dose VC can reduce myocardial dysfunction in septic rats induced by CLP, but there are few studies on the mechanism of action of VC in sepsis.

Inflammation and apoptosis are recognized as key pathophysiological phenomena in sepsis and SIMI at the cellular and molecular levels [7]. Many basic studies have indicated that regulate inflammation and apoptosis can reduce infectious cardiac insufficiency [35, 36]. Some studies have shown that VC could attenuate inflammation, oxidative damage, and apoptosis [37, 38]. However, whether high-dose VC can alleviate CLP-induced SIMI by inhibiting suppressing inflammatory and apoptosis has not been studied at different points in time. TUNEL staining and expression of Cleaved-Caspase 3, Bax, Cleaved-Caspase 9, and Bcl-2 confirmed that VC significantly reduced cardiomyocyte apoptosis. Although at some point in time the protein expression did not achieve the desired effect, which may be the result of manipulation discrepancies or insufficient sample sizes, we were still able to generate the conclusion that high-dose VC treatment had a positive effect on the overall trend of CLP-induced cardiomyocyte apoptosis.

During the occurrence and development of sepsis, over-activated inflammatory response is an important reason for the aggravation of the disease [39–43]. MAPK and NF-κB signaling pathways were activated during SIMI and regulated inflammatory response and apoptosis [44–46]. Li et al. [47] found that Pinocembrin can reduce inflammatory damage by inhibiting p38/JNK MAPK signaling pathway, thus achieving the therapeutic effect of SIMI. In addition, Zhang et al. [48] found that L-carnitine could inhibit inflammation and apoptosis by regulating MAPK signaling pathway, thus achieving the therapeutic effect of SIMI. At the same time, our previous studies also showed that inhibiting inflammation and apoptosis by inhibiting NF-κB signaling pathway can also achieve the purpose of SIMI treatment [9]. Our study was consistent with that, the levels of p-JNK, p-Erk1/2, p-P38, p-NF-κB and p-IKK α/β were higher in sepsis and SIMI, indicating that MAPK and NF-κB were activated, while high-dose VC reversed their phosphorylation. In sepsis, studies have found that both anti-inflammatory and pro-inflammatory cytokines are elevated in the early stage, and these inflammatory factors are helpful in judging the prognosis of sepsis [49]. The three major pro-inflammatory cytokines TNF-α, IL-1β and IL-6 were not specific for sepsis and their primary role as biomarkers of sepsis appears to be prognostic rather than diagnostic [50]. It’s been found in patients with sepsis that those who die often have significantly elevated expression of inflammatory cytokines [51]. In critically ill hospitalized patients, the elevation of IL-10 is very significant at an early stage [52]. In our study, the levels of IL-6 and IL-10 were also obviously higher in the CLP-induced group, which indirectly explains the high mortality in CLP-induced rats. And the levels of IL-1β and TNF-α were also importantly higher in the CLP-induced group. But high-dose VC significantly reversed the levels of TNF-α, IL-1β and IL-6, while raising anti-inflammatory cytokines IL-10 levels instead of lowering them. The plausible explanation for VC increasing IL-10 levels rather than decreasing them should be that IL-10 can reduce the production and activity of pro-inflammatory cytokines, thereby regulating the inflammatory response [53]. Our results suggest that high-dose VC can protect SIMI by regulating NF-κB and MAPK signaling pathways to inhibit apoptosis and inflammation.

Sepsis induces autophagy in multiple organs [54–56], including the heart [57–59]. Investigations *in vivo* using a CLP sepsis model [60] and *in vitro* using cultured, LPS-induced cardiomyocyte injury [61] have showed that stimulation of autophagy can improve cardiac function, thus suggesting that autophagy pharmacologically protected the myocardium. Other studies have shown autophagy increased in the early stages of sepsis and subsequently decreased near advanced organ failure [62, 63]. Several basic studies [64–66] proposing various therapeutic approaches have identified improvements in SIMI by mediating autophagy. Beclin 1 [67], LC3 [68] and P62 [69] have become markers of autophagy activity in current studies [70, 71]. In this study, LC3-II/LC3-I ratio and the expression levels of Beclin-1 in CLP-induced SIMI significantly decreased, while P62 significantly increased, indicating that autophagy was attenuated in SIMI and autophagy inhibition was linked to the onset and progression of CLP-induced SIMI. However, high-dose VC treatment reversed their results, which might be due to enhanced autophagy by high-dose VC treatment, thus protecting against SIMI. Therefore, a potential strategy to alleviate CLP-induced SIMI might be high-dose VC’s promotion of autophagy to eliminate cardiomyocyte inflammatory and apoptosis.

Next, we further investigated the anti-apoptotic, anti-inflammatory, and pro-autophagic cardioprotective effects of high-dose VC in sepsis, thereby exploring its potential protective mechanism against CLP-induced SIMI. The PI3K/AKT/mTOR has recently been shown to be a key pathway in the regulation of autophagy, inflammation, and apoptosis [72, 73]. In addition, the regulation of PI3K/AKT/mTOR pathway was closely related to the regulation of MAPK and NF-κB pathways. The PI3K/AKT signaling pathway plays a pro-inflammatory and anti-inflammatory role by activating NF-κB downstream of AKT to promote the production of pro-inflammatory cytokines [74, 75]. Some studies [76] found that after both MAPK and PI3K/AKT signaling pathways were activated by LPS, MAPK signaling was more prominent than PI3K/AKT signaling, which further increased apoptosis. Studies have shown that Beclin-1 signal activation can inhibit mTOR [77], enhance AMPK [78], and alleviate cardiac inflammatory injury during LPS-induced myocardial injury, indicating that Beclin-1 dependent autophagy plays a protective role in SIMI [61]. In our study, the phosphorylation of mTOR was consistently enhanced at three time points during SIMI, while the expression of mTOR phosphorylation was decreased after high-dose VC treatment. Therefore, it is speculated that high-dose VC treatment enhances autophagy and inhibits the activation of mTOR. What’s more, in the treatment of sepsis, the PI3K/AKT/mTOR signaling pathway was described in two ways. The first one was that activation of PI3K/AKT/mTOR signaling pathway can improve sepsis-induced cardiac dysfunction [79, 80]. However, other studies have found that inhibition of PI3K/AKT signaling pathway has a protective effect on SIMI [76]. The difference in results may be related to the severity of the disease at the time of study and the duration of treatment. Meanwhile, blocking the PI3K/AKT/mTOR signaling pathway during sepsis triggers autophagy and blocks apoptosis [7]. A recent study showed that VC could protect against SIMI by inhibiting AKT/mTOR pathway activation and pyrodeath [81]. The present study discovered that the levels of p-AKT, p-PI3K and p-mTOR were elevated in SIMI rats, whereas high-dose VC reduced their phosphorylation levels, indicating that high-dose VC protected SIMI in rats by the inhibition of PI3K/AKT/mTOR signaling pathway, consistent with the latter view. Sum up, high-dose VC may potentially result in inhibition of the PI3K/AKT/mTOR pathway in sepsis, reducing myocardial apoptotic damage and inflammatory storm, increasing autophagy, and ultimately protecting the myocardium.

In this study, SIMI was observed at 1d, 3d and 5d after CLP operation, and the treatment of SIMI was observed after high-dose VC treatment. Although these studies have important findings, there are limitations. It should be noted that we did not further investigate how high-dose VC affects myocardial apoptosis, inflammation and autophagy during sepsis through MAPK, PI3K/AKT/mTOR and NF-κB pathways.

## CONCLUSION

**Figure 7:**
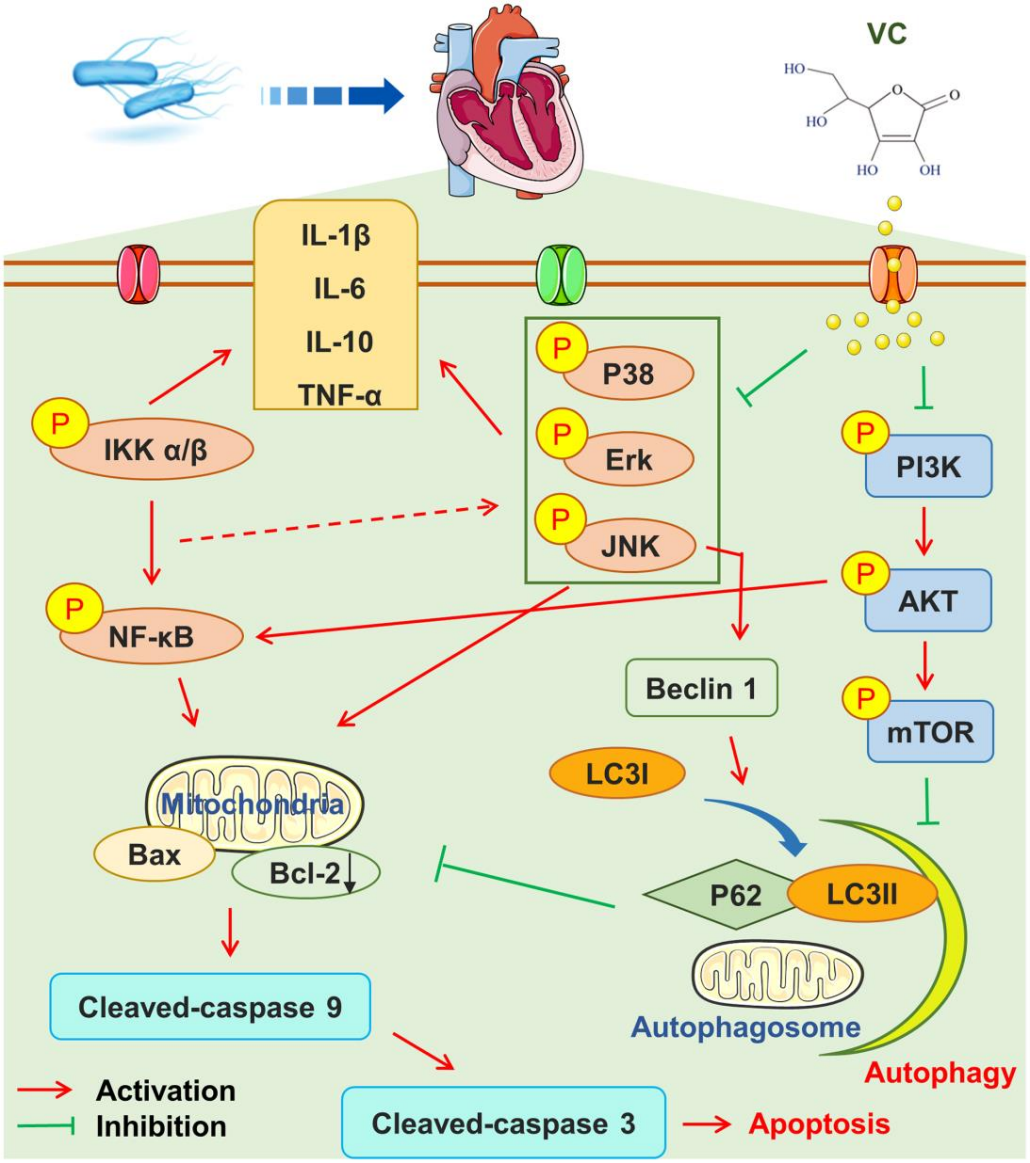
High-dose VC could mediate by inhibiting apoptosis and inflammatory, and promoting autophagy through regulating MAPK, NF-κB and PI3K/AKT/mTOR pathway.

To sum up, CLP-induced myocardial injury and dysfunction were found to be time-dependent. In addition, our found that high-dose VC can resist CLP-induced SIMI and protect myocardial cells by inhibiting apoptosis, inhibiting inflammation and enhancing autophagy mediated by MAPK, PI3K/AKT/mTOR and NF-κB signaling pathways (Figure 7). Therefore, we provide strong support for the putative mechanism of high-dose VC against CLP-induced SIMI, and also provide basic research evidence for the clinical use of high-dose VC in the treatment of sepsis.

## MATERIALS AND METHODS

### Animal experiment

Sprague Dawley (SD) rats were randomly divided into 3 groups (*n* = 18/group) (sham group, CLP group and VC group). Each group of rats was randomly divided into 1 d, 3 d and 5 d group, which were treated with drug for 1 d, 3 d and 5 d, respectively. At 1 hour after CLP or sham operation, the rats in VC group were given VC (Shandong Xinhua Pharmaceutical Co., Ltd. 500 mg/kg i.v.) by intravenous infusion, and the rats in sham operation group or CLP group were given the same amount of normal saline. The CLP surgical procedure was consistent with our previous study [7]. The entire process is shown in Figure 1A.

### Survival experiment

According to the method of grouping in the previous experiment, the survival analysis experiment consisted of 10 rats per group. The surgical procedure is the same as the drug regimen. After the operation, the survival of rats was observed every 12 hours for a maximum of 7 days.

### Echocardiography, H&E staining, ELISA, TUNEL staining and biochemical detection

Echocardiography, H&E staining, TUNEL staining, ELISA (IL-1β, IL-6, IL-10 and TNF-α) and Biochemical Detection (cTnI, CK and LDH) assay were performed on days 1, 3, and 5, respectively. Details are provided in a previously published article [7].

### Western blot

The experimental procedures for total protein sample preparation, protein concentration detection, electrophoresis, membrane transfer, and blocking were as described in previous publications from our research group. Membranes were incubated with primary antibodies followed by secondary antibodies. The primary antibodies used were: P38 (cat. AF6456, 1:1000), Erk1/2 (#4695, 1:1000), p-P38 (cat. AF4001, 1:1000), p-Erk1/2 (#9101, 1:1000), JNK (cat. AF6318, 1:1000), NF-κB (cat. AF5006 1:1000), p-JNK (cat. AF3318, 1:1000), p-NF-κB (cat. AF2006 1:1000), p-IKK α/β (cat. AF3013 1:1000), PI3K (cat. AF6241, 1:1000), phosphorylated (p)-PI3K (cat. AF3241, 1:1000), AKT (cat. AF6261, 1:1000), p-AKT (cat. AF0016, 1:1000), Cleaved-caspase 9 (cat. AF5240, 1:1000), mTOR (cat. AF6308, 1:1500), P62 (cat. Ab91526, 1:1000), p-mTOR (cat. AF3308, 1:1500), Bax (cat. AF0120, 1:2000), Bcl-2 (cat. AF6139, 1:2000), Cleaved-caspase 3 (cat. AF7022, 1:1000), Caspase 3 (cat. AF6311, 1:1000), Beclin 1 (cat. Ab62557, 1:1000), GAPDH (cat. T0004, 1:10,000) or LC3-I/II (cat. Ab128025, 1:1000). GAPDH was used as the control for sample loading and integrity. p-Erk1/2 and Erk1/2 antibodies were purchased from Cell Signaling Technology in America. All other antibodies were purchased from Affinity in China. Finally, the corresponding secondary antibodies (goat anti-rabbit (cat. S001, 1:10,000) or goat anti-mouse (cat. AS014; 1:10,000, Abclonal)) were incubated according to the properties of the primary antibodies and exposed with ECL luminescent solution. ImageJ software was used for analysis.

### Immunohistochemistry (IHC)

4 μm thick sections in HE were incubated with primary antibodies (p-AKT (Ser473, cat. AF0016, dilution, 1:100) and p-PI3K (Tyr607, cat. AF3241, dilution, 1:100)), and Goat anti-Rabbit lgG (cat. ZF-0516, 1:100) secondary antibodies were then incubated. Sections were then treated with streptavidin-horseradish peroxidase (DF7852, Shanghai Yaoyin Biotechnology Co., Ltd., Shanghai, China), stained with diamine benzidine and counterstained with hematoxylin.

### Statistical analysis

All data were processed using SPSS 24.0 statistical software. The measured data are presented as mean ± SD (*n* = 6/group). The data between the two groups were analyzed by independent sample *t*-test. If the *p*-values were < 0.05, then the differences were considered statistically significant. The one-week survival rate was calculated and analyzed by Kaplan-Meier.
